# Dissolution Behavior of Flufenamic Acid in Heated Mixtures with Nanocellulose

**DOI:** 10.3390/molecules25061277

**Published:** 2020-03-11

**Authors:** Athanasios Mantas, Albert Mihranyan

**Affiliations:** Nanotechnology and Functional Materials, Department of Materials Science and Engineering, Box 534 Uppsala University, 75121 Uppsala, Sweden; athanasios.mantas@angstrom.uu.se

**Keywords:** fenamates, fasted/fed state variability, microcrystalline cellulose, *Cladophora* cellulose, polymorphism

## Abstract

Flufenamic acid (FFA) is a problem drug that has up to eight different polymorphs and shows poor solubility. Variability in bioavailability has been reported in the past resulting in limited use of FFA in the oral solid dosage form. The goal of this article was to investigate the polymorphism and amorphization behavior of FFA in non-heated and heated mixtures with high surface area nanocellulose, i.e., *Cladophora* cellulose (CLAD). As a benchmark, low surface area microcrystalline cellulose (MCC) was used. The solid-state properties of mixtures were characterized with X-ray diffraction, Fourier-transform infrared spectroscopy, and differential scanning calorimetry. The dissolution behavior of mixtures was studied in three biorelevant media, i.e., fasted state simulated gastric fluid, fasted state simulated intestinal fluid, and fed state simulated intestinal fluid. Additional thermal analysis and dissolution tests were carried out following 4 months of storage at 75% RH and room temperature. Heated mixtures of FFA with CLAD resulted in complete amorphization of the drug, whereas that with MCC produced a mixture of up to four different polymorphs. The amorphous FFA mixture with CLAD exhibited rapid and invariable fasted/fed state dissolution in simulated intestinal fluids, whereas that of MCC mixtures was highly dependent on the biorelevant medium. The storage of the heated FFA-CLAD mixture did not result in recrystallization or changes in dissolution profile, whereas heated FFA-MCC mixture showed polymorphic changes. The straightforward dry powder formulation strategy presented here bears great promise for reformulating a number of problem drugs to enhance their dissolution properties and reduce the fasted/fed state variability.

## 1. Introduction

Enhancement of solubility of poorly soluble drugs is one of the most intensively investigated topics within the pharmaceutical formulation science today. The issues of solubility-limited bioavailability can be further complicated by polymorphism and solid-state instability, presenting great challenges for the industry. Therefore, extensive studies on relatively challenging molecules are required to develop new formulation strategies that can both enhance the bioavailability of the drug and reduce the inter-individual variability. The present study focuses on the formulation of flufenamic acid (FFA) as a model poorly soluble drug with high degree of polymorphism. In particular, as many as nine polymorphs have been described for FFA [1].

Flufenamic acid, together with mefenamic acid (MFA) and tolfenamic acid (TFA), belongs to the class of fenamates, which were developed in the 1960s [2]. Being among the top prescribed NSAIDs in the 1990s, the prescription rate of fenamates has declined during the last decade, although it is still significant in Asian countries [3]. FFA was never registered in the USA, and it is currently rarely used in oral solid dosage forms, albeit it may find use in topical formulations. Compared to the most common fenamate on the market, i.e., MFA, FFA would normally be administered at much lower doses, i.e., 100 mg vs. 250 mg, respectively [4].

Traditional uses of FFA and other fenamates include analgesic treatment of rheumatoid arthritis, migraine, and, especially, primary dysmenorrhea. The distinctive utility in dysmenorrhea treatment has been linked to a dual mode of action involving not only inhibition of prostaglandin synthases, but also antagonism to prostaglandin itself [4]. Because of the dual action mode, pretreatment is not required, and dosage can be started as soon as the first dysmenorrhea symptoms commence, e.g., with the first menstrual flow or spasmodic pain [5]. Furthermore, fenamates not only relieve pain associated with dysmenorrhea, but also significantly reduce menorrhagia and craving for other analgesics during the treatment [4,5,6]. Following FFA treatment, it was reported that 82% of patients (n = 36) experienced significant relief of pain, 66%—reduced episodes of vomiting, and 52%—reduced incidence of diarrhea [7]. Lately, FFA received renewed interest for new indications, such as ion channel modulators. [8] It should, however, be noted that fenamates show higher incidence of gastrointestinal side effects (30–60%) compared to OTC NSAIDs, e.g., ibuprofen or naproxen [2].

High inter-individual variability of pharmacokinetic parameters in humans has been cited as one of the main limiting factors for FFA’s practical use [9]. Although fenamates exhibit high absorption rates (up to 80%) following oral administration, variable bioavailability was reported for FFA with peak plasma concentrations ranging between 6 and 20 μg/mL, which were reached between 1.5 and 5 h [9]. Concomitant food intake with FFA was reported to affect the drug uptake, varying by up to 30% depending on the formulation [10]. Furthermore, highly variable FFA bioavailability was reported for 5 different formulations in vivo highlighting FFA’s reputation of a problem drug [11].

We have previously shown that heat-assisted mixing of poorly soluble drugs from varying pharmacological classes with high surface area nanocellulose results in enhanced solubility and dissolution rate in biorelevant media. In particular, the latter effect was shown for NSAIDs from arylpropionic acid derivatives, e.g., ibuprofen, flurbiprofen, ketoprofen, and naproxen [12], as well as dihydropyridine-type calcium channel blockers, e.g., nifedipine, felodipine [13]. In this article, we extend this strategy and demonstrate improved formulation for a model problem drug featuring both poor solubility and unusually high number of polymorphs. In particular, we demonstrate enhanced dissolution rate and reduced dissolution variability between the fed and fasted states of FFA. The latter effect is achieved by amorphization of FFA in the dry mixtures with high surface area nanocellulose, i.e., *Cladophora* cellulose (CLAD). The formulation of FFA with low surface area cellulose, i.e., microcrystalline cellulose (MCC), does not achieve a similar effect.

## 2. Materials and Methods

### 2.1. Materials

*Cladophora* cellulose was provided by FMC Biopolymers (currently DuPont, Philadelphia, PA, USA). Microcrystalline cellulose (Avicel PH101) and flufenamic acid (FFA) were purchased from Sigma Aldrich (St. Louis, MO, USA). Biorelevant media of simulated gastric fluid (SGF), fasted state simulated intestinal fluid (FaSIF) and fed state simulated intestinal fluid (FeSIF) were prepared using the powder purchased from Biorelevant (London, Great Britain) according to the manufacturer’s instructions.

The structure and physicochemical properties of FFA are presented in Table 1. 

### 2.2. Materials Preparation

#### 2.2.1. Physical (Normal) Mixture (P)

FFA powder was used as supplied without sieving to separate a specific particle size fraction. The mixture was prepared by dry blending of FFA with cellulose. The weight ratio between FFA and cellulose was 1:9. Typically, 5 mg of FFA was mixed with 45 mg of cellulose in a glass vial using a Turbula mixer (Muttenz, Switzerland) for 15 min.

#### 2.2.2. Heated Mixture (H)

Following preparation of physical mixtures, the sealed vials were heated to 134 °C for 1 h.

### 2.3. Materials Characterisation

#### 2.3.1. Scanning Electron Microscopy (SEM)

SEM images of cellulose powder samples were acquired with a scanning electron microscope (Merlin FEG-SEM, Zeiss, Germany). The membranes were sputtered with Au/Pd prior to analysis to minimize charging effects of the samples. A Polaron sputter coater (Ashford, UK) was used. The sputtering settings were 4 × 10^−2^ mbar and 35 mA, and the sputtering time was 30 s.

An accelerated stability study was conducted on FFA-cellulose mixtures to follow solid-state transitions. Glass vials containing the heated sample were stored at constant relative humidity (75%) over a saturated NaCl solution at room temperature for 4 months in a desiccator.

#### 2.3.2. Powder X-Ray Diffraction (PXRD)

An X-ray diffractometer (D8 Twin-Twin, Bruker, Karlsruhe, Germany) with Bragg−Brentano geometry (CuKα radiation; λ = 1.54 Å) was used. Operating current settings were 40 kV and 40 mA. The 2θ angle was varied between 10° and 45° at 0.02° scan steps. The data were collected on flat powders placed in reduced background specimen holders supplied by the manufacturer (Bruker, Karlsruhe, Germany).

#### 2.3.3. Differential Scanning Calorimetry (DSC)

The DSC measurements were performed with a Mettler Toledo DSC 3 instrument (Mettler Toledo, Schwerzenbach, Switzerland). The samples were first cooled from room temperature to −30 °C, and then heated to 160 °C at the 10 K/min heating rate. Constant nitrogen gas at a flow rate of 60 mL/min was purged throughout the measurements. Typically, 1 mg of 1:9 drug-cellulose mixture was used per measurement. For pure substances, 1 mg of FFA and 1 mg of cellulose were used per measurement. The aluminum pan containing the sample was punctured to avoid overpressure. The collected data were treated with the eStar analysis software provided by Mettler Toledo.

Crystallinity index was calculated as follows:

(1) $$CrI=\;\left(\frac{∆H_{mixture}}{∆H_{drug}\times a}\right)\times 100$$

where Δ*H_mixture_* is the enthalpy of FFA melting in the mixture, Δ*H_drug_* is the melting enthalpy of a pure drug, and a is the correction factor corresponding to drug content, i.e., a = 1 for a pure drug, and a = 0.1 for a 10% *w*/*w* mixture.

#### 2.3.4. Fourier-Transform Infrared Spectroscopy (FTIR)

FTIR absorbance spectrum was acquired to follow the interactions between FFA and cellulose. The most informative region relevant for this study included the area from 1800 to 1400 cm^−1^ corresponding to the stretch of C=O bonds. The measurements were performed with a Bruker Tensor 27 FTIR (Billerica, MA, USA) according to the pellets technique with potassium bromide (KBr). The amount of FFA in the drug-cellulose mixture (1:9 *w*/*w*) was about 2 mg. The amount of KBr used was around 200 mg. The collected data were treated with the OPUS Data Collection Program (V 1.1).

#### 2.3.5. In Vitro Dissolution Test in Biorelevant Media

The dissolution test was performed in a SOTAX (AT7 Smart, Basel, Switzerland) apparatus using 500 mL of the biorelevant medium per dissolution vessel. The temperature for each dissolution vessel was maintained at 37 °C. The same amount of mixed samples was used in all timings and all different media (100 mg FFA + 900 mg cellulose). Each group of non-heated and heated samples was run in triplicate. The paddle speed for each dissolution vessel was 50 rpm. The sampling times for all media were 5, 15, 30, 45, 60, 90, and 180 min, respectively. At each time point, 5 mL of the medium was withdrawn and filtered through a 0.45-μm PTFE filter after discarding the first 2–3 mL. A total of 1.5 mL of the remaining sample was transferred in glass vials for further analysis by HPLC.

#### 2.3.6. High Performance Liquid Chromatography (HPLC)

A Hitachi Chromaster HPLC-UV system was optimized for the detection of FFA prior to the analysis. The liquid chromatography system used was a Hitachi Chromaster pump 5110 with a Hitachi Chromaster 5260 autosampler and Purospher^®^ STAR RP-18e (2 µm) Hibar^®^ HR 50–2.1 mm column (Merck, Darmstadt, Germany). The column temperature was 50 °C, and injector temperature was 20 °C. The mobile phase A was 0.1% formic acid in water and mobile phase B was 0.1% formic acid in acetonitrile. The flow rate was 0.8 mL/min. The injector wash was 50% acetonitrile. The retention time was 4.45 min, and run time was 8.0 min. UV detection at the wavelength of 280 nm was used. Calibration samples were run prior to the analysis of the study samples by preparing the FFA stock solution in the biorelevant medium at a concentration of 250 μg/mL. The stock solution was used as the calibration standard by a subsequent gradual dilution of 1:250.

## 3. Results

The cellulose batches used in this study were identical to those used previously [12,13]. Notably, the pore volume of CLAD was 276 times and the specific surface area of CLAD was about 108 times larger than that for MCC as derived from N_2_ gas sorption analysis.

### 3.1. Scanning Electron Microscopy (SEM)

**Figure 1:**
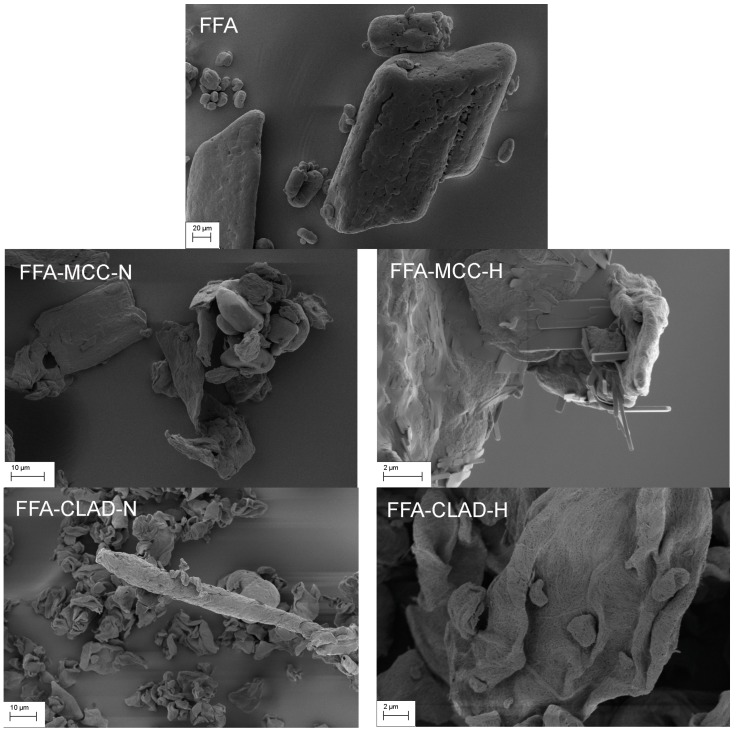
SEM images of the pure drug and powder mixtures, i.e., of pure FFA (upper panel), FFA-MCC-N (middle left panel), FFA-MCC-H (middle right panel), FFA-CLAD-N (lower left panel), and FFA-CLAD-H (lower right panel).

Figure 1 shows the SEM images of the pristine FFA drug and FFA-cellulose mixtures. Particles of varying size could be seen in the pristine FFA. FFA particles featured distinct morphology with rounded edges. In FFA-MCC-N mixtures, the pristine FFA crystals were clearly visible next to the MCC particles. However, in the heated FFA-MCC sample, the morphology of FFA was significantly altered. In particular, the surface of MCC particles appeared to be covered with elongated slab-like particles. The particle size of the slab-like elongated particles was in the submicron range, which was at least one order of magnitude smaller than that of the pristine FFA particles. It should be noted that no FFA particles with rounded edges were detected in SEM images of FFA-MCC-H. Figure 1 shows the SEM images of the studied samples. Normally, individual CLAD particles feature a raisin-like wrinkled shape. Interestingly, atypical rod-like structures apparently produced by woven individual CLAD particles were detected in SEM following dry mixing in the FFA-CLAD-N mixture. Typical round-edged FFA crystals were difficult to spot, although one such crystal could be seen behind the woven rod-like structure in the presented image. Figure 1 shows the SEM image of the FFA-CLAD-H sample. Unlike the heated FFA-MCC sample, no slab-like elongated particles could be seen on the top of the CLAD particles. In fact, no FFA particles could be detected, and the texture of CLAD particles largely resembled that of pure CLAD cellulose.

### 3.2. Powder X-Ray Diffraction (PXRD)

**Figure 2:**
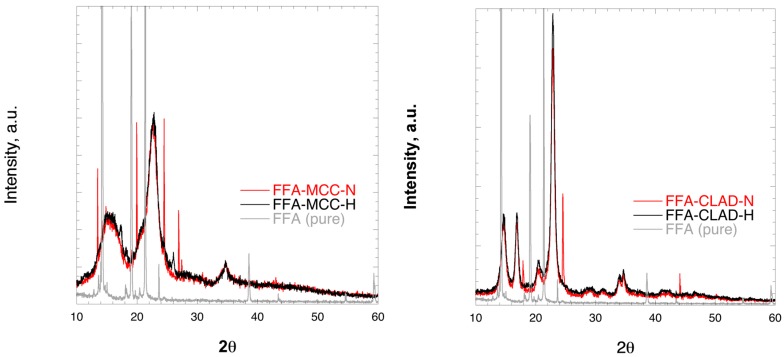
X-Ray diffraction images of the pure drug and powder mixtures, i.e., FFA-MCC (left panel), and FFA-CLAD (right panel); a.u.= arbitrary units.

Figure 2 shows the XRD profiles of the FFA-MCC and FFA-CLAD mixtures. The characteristic peaks for crystalline FFA were clearly observed in the physical mixture of FFA-MCC-N, and in FFA-CLAD-N mixtures. However, they were substantially suppressed in FFA-MCC-H or completely disappeared in the FFA-CLAD-H mixture. It is interesting to note that the position of the visible crystalline peaks in the cellulose mixtures was shifted to different 2θ angles than those of the pure FFA used as the starting material. It is known that FFA can form different polymorphs, which could explain the molecular rearrangement.

### 3.3. Differential Scanning Calorimetry (DSC)

**Figure 3:**
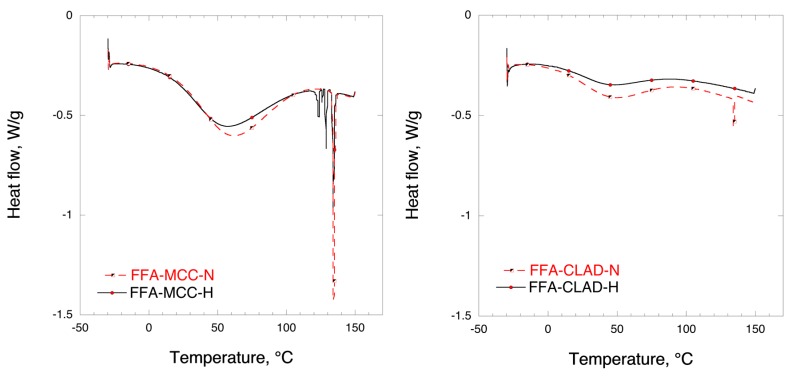
Differential scanning calorimetry (DSC) profiles of FFA-MCC (left panel), and FFA-CLAD (right panel) 10% *w*/*w* mixtures.

Figure 3 shows the DSC results for the mixtures of FFA with MCC and CLAD. In the DSC profile, two events can be distinguished, i.e., water evaporation and melting of FFA. The broad hallow at temperatures < 100 °C represents the water evaporation enthalpy. The endothermic event due to FFA melting is clearly visible around 134 °C in the profiles for FFA-MCC-N and FFA-CLAD-N, confirming the results of XRD. It should be noted that the enthalpy of the non-heated FFA melting peak in the FFA-CLAD-N mixture is substantially lower than that of FFA-MCC-N, suggesting that some solid-state rearrangement occurred in the non-heated CLAD sample during mixing. In the FFA-MCC-H mixture, several endothermic events are visible. These sharp endothermic peaks are likely to be due to FFA polymorphs formed during recrystallization. No melting endotherm was observed for FFA-CLAD-H mixture. The latter suggests that FFA is in the amorphous state, which is corroborated by the XRD analysis data.

Table 2 summarizes the results of DSC analysis for the studied samples. It is seen in this table that the degree of FFA crystallinity in the non-heated CLAD mixture is only 11.9%, whereas in the non-heated FFA-MCC-N mixture it is 97.2%. The predominant crystalline form in the FFA-MCC-H sample is crystalline FFA with the melting point of 134 °C, i.e., polymorph A, which stands for nearly half of the crystallinity index. In total, there are 4 different polymorphs detected with cumulative crystallinity index of 87.7%. The latter suggests that nearly all FFA recrystallized into different polymorphic forms after heating. For comparison, essentially all FFA is in the amorphous state in the heated CLAD sample.

### 3.4. Fourier-Transform Infrared Spectroscopy (FTIR)

**Figure 4:**
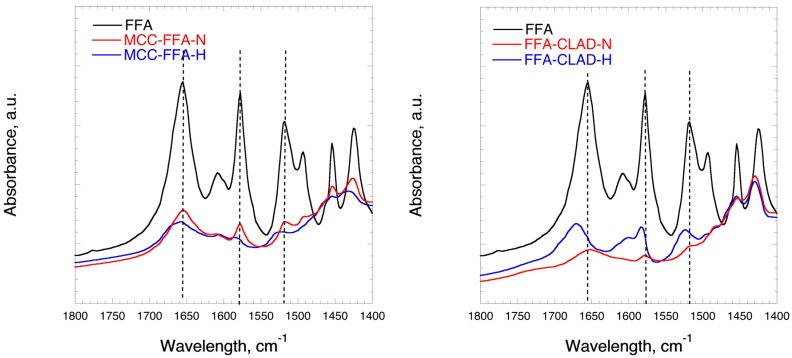
FTIR profiles of the pure drug, FFA-MCC-N and FFA-MCC-H (left panel), and FFA-CLAD (right panel) 10% wt mixtures.

Figure 4 shows the FTIR profiles of FFA in mixtures with celluloses in the regions corresponding to the stretch of C=O bonds. In the FFA-MCC-H mixture, no shift in the position of the C=O bond at 1651 cm^−1^ is seen as compared to the FFA-MCC-N sample. On the other hand, in the FFA-CLAD-H mixture, a clearly visible shift of the stretch of C=O bonds is observed from 1651 cm^−1^ to 1670 cm^−1^ as compared to the non-heated mixture. The position of the C=O bond at 1651 cm^−1^ in the normal mixture normally coincides with that of the pure FFA.

Based on the XRD, DSC, and FTIR results, it can be concluded that heated mixtures of FFA with CLAD show significant degree of FFA molecular rearrangement and render the latter amorphous. In the previous studies, amorphization of poorly soluble drugs in mixtures with CLAD resulted in enhanced solubility and dissolution rate.

### 3.5. In Vitro Dissolution in Biorelevant Media

**Figure 5:**
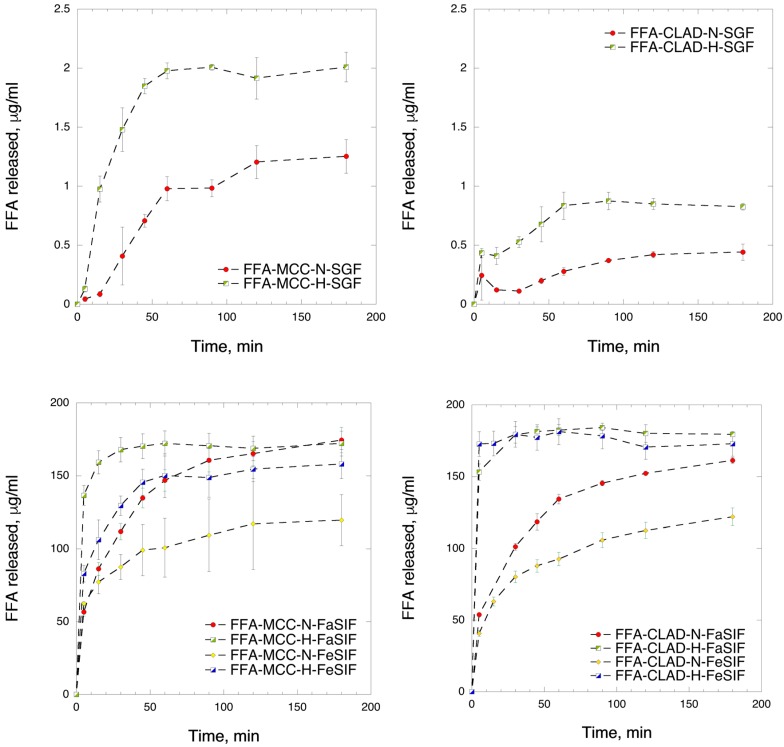
In vitro time-dependent FFA dissolution in biorelevant media, i.e., FFA-MCC mixtures in SGF medium (upper left panel); FFA-CLAD mixtures in simulated gastric fluid (SGF) medium (upper right panel); FFA-MCC mixtures in fasted-state simulated intestinal fluid (FaSIF) and fed-state simulated intestinal fluid (FeSIF) media (lower left panel); and FFA-CLAD mixtures in FaSIF and FeSIF media (lower right panel). The results are the average of three measurements, with the standard deviation shown as error bars. The dashed line is added as a visual guide.

Figure 5 shows the FFA dissolution profiles in different biorelevant media. The maximum solubility observed in biorelevant intestinal media is 180 μg/mL, which corresponds to 90% of drug loading, whereas that in SGF is 2 μg/mL, i.e., only 1% of the loading. Hence, the solubility of FFA is the lowest in SGF, which is expected based on its chemical structure. It is seen in the graph that the release from FFA-MCC samples was higher than that from FFA-CLAD, even though the degree of FFA crystallinity was significantly higher in the former samples. In general, the release from FFA-MCC samples was nearly 50% higher than that from FFA-CLAD in SGF. This could be due to stronger association with the high surface area CLAD cellulose, or due to FFA molecules sitting deeper inside the pores of CLAD.

Figure 5 shows the release data for MCC mixtures in FaSIF and FeSIF media. It is seen in the graph that the FFA release rate from the heated mixtures was enhanced compared to the non-heated mixtures. However, it should be noted that there is discrepancy between the rate of release for FaSIF and FeSIF media for each respective mixture. From the point of poorly soluble drug formulation, the fasted/fed state variability is undesirable. Figure 5 shows the release from CLAD mixtures. It is seen from the graph that heated FFA-CLAD-H samples exhibit markedly improved dissolution profiles compared to the non-heated mixtures in both FeSIF and FaSIF. In particular, within the first 5 min, FFA concentration reaches a plateau, whereas that for the non-heated sample reaches a similar level only after 3 h of dissolution in a FaSIF. The dissolution of FFA-CLAD-N in a FeSIF is very slow and does not reach the maximum level even after 3 h of dissolution. More importantly, the fasted/fed state variability is dramatically decreased, which contrasts starkly the results observed for FFA-MCC-H data.

We reported previously that the amorphization of poorly soluble aromatic pharmaceutical compounds in high surface area CLAD cellulose is sufficiently stable over time to enable practical applications. The solid-state stability for high challenging polymorphic substances therefore needs to be elucidated. The produced mixtures were stored at 75% RH and room temperature for over 4 months. Following the storage, the mixtures were studied with DSC to monitor phase transitions. Furthermore, the FFA release was re-evaluated after storage in all 3 biorelevant media.

### 3.6. Differential Scanning Calorimetry (DSC) (After Storage)

**Figure 6:**
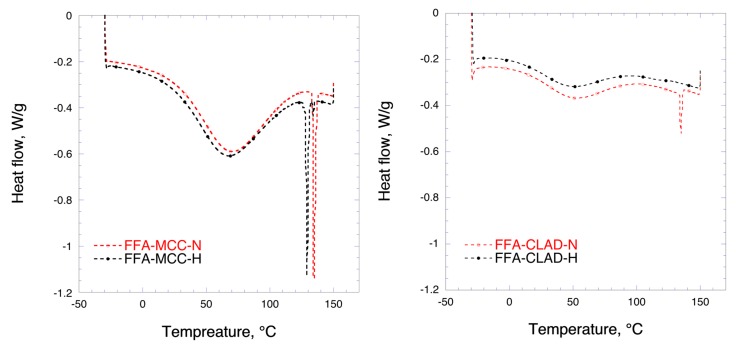
Differential scanning calorimetry (DSC) profiles of FFA-MCC (left panel), and FFA-CLAD (right panel) 10% *w*/*w* mixtures.after storage.

Figure 6 shows the DSC profiles of the mixtures after storage. No significant phase transition was observed in the FFA-MCC-N sample following the storage. On the other hand, the DSC profile of the FFA-MCC-H sample after 4 months of storage showed presence of two endothermic peaks at around 129 and 134 °C, respectively, as opposed to 4 polymorphs observed in the pristine FFA-MCC-H. Furthermore, the predominant type of FFA was polymorph B (123 °C), rather than polymorph A (134 °C). The latter suggests recrystallization and phase transitions occurring in the FFA-MCC-H sample when stored at high RH. The DSC profiles of FFA-CLAD mixtures were essentially unchanged following the storage at 75% RH and room temperature. Most importantly, no recrystallization was observed in the amorphous FFA-CLAD-H sample upon storage. Table 3 summarizes the results of the DSC analysis.

### 3.7. In Vitro Dissolution in Biorelevant Media (After Storage)

**Figure 7:**
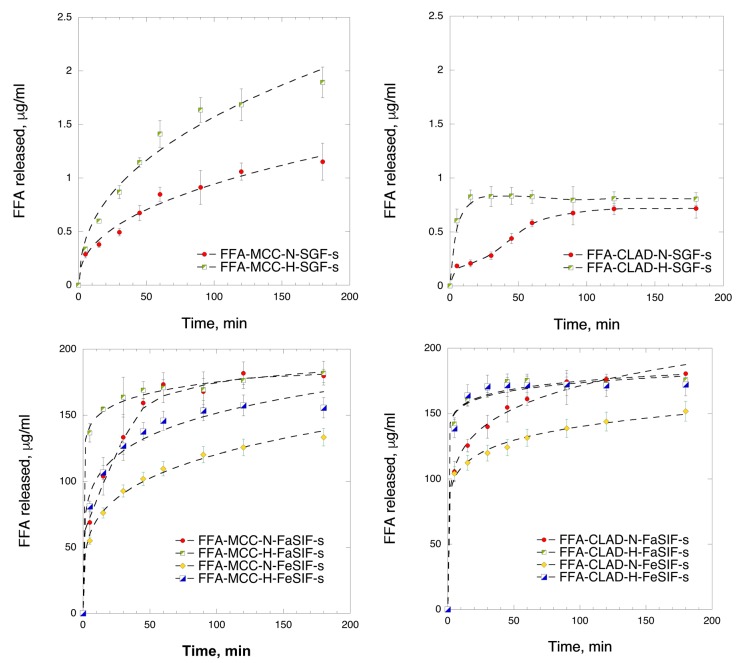
In vitro time-dependent FFA dissolution in biorelevant media, i.e., FFA-MCC mixtures in SGF medium (upper left panel); FFA-CLAD mixtures in SGF medium (upper right panel); FFA-MCC mixtures in FaSIF and FeSIF media (lower left panel); and FFA-CLAD mixtures in FaSIF and FeSIF media (lower right panel) after storage. The results are the average of three measurements, with the standard deviation shown as error bars. The dashed line is added as a visual guide.

Figure 7 shows the in vitro dissolution profiles of the studied samples after storage. The drug release experiments in biorelevant media following the storage largely corroborated the previous observations. In the SGF medium, the release from the MCC samples was higher than that from the CLAD samples. However, the rate of release was slightly slower in the respective FFA-MCC mixtures after storage compared to the pristine mixtures. The patterns observed in the FaSIF/FeSIF media after storage were also similar to those in the pristine mixtures. In particular, high variability in the rate of dissolution was observed between FaSIF and FeSIF media for the FFA-MCC-H samples. On the contrary, the release from FFA-CLAD-H in these media was very rapid and did not exhibit variability between the media. The latter is ascribed to the stable amorphization of FFA in the heated CLAD mixture.

The results of the present study confirm the previous conclusions on the usefulness of high surface area nanocellulose to induce and maintain amorphization of poorly soluble aromatic pharmaceutical compounds, which was previously observed for profens, e.g. ibuprofen, and nifedipine. That the observed effect can be maintained for a highly challenging polymorphic substance is novel. The underlying mechanism for this effect is most likely the interplay between H-bonding and hydrophobic (π-π and π-OH) interactions between cellulose and drug molecules, which has been confirmed by molecular dynamics studies [14]. The straightforward dry powder formulation strategy presented here bears great promise for reformulating a number of problem drugs to enhance their dissolution properties and reduce the fasted/fed state variability.

## 4. Conclusions

The results of the present study show that the heated mixtures of FFA-CLAD produce stable amorphous mixtures which exhibit enhanced dissolution rate and reduced fasted-fed state variability in biorelevant intestinal media. Heated FFA-MCC mixtures, i.e., with low surface area, do not produce a similar amorphization effect, especially with respect to the fasted-fed state variability. Instead, FFA recrystallizes into 4 different polymorphs rather than stable amorphous state as is the case with the FFA-CLAD-H sample. The different polymorphic entities and variable dissolution profile result in unpredictable dissolution behavior of FFA in MCC mixtures. The solubility of FFA in the SGF is very low, and ≤ 1% of the loaded drug, i.e., ≤ 2 μg/mL, is dissolved. The amount of FFA dissolved from MCC sample in the SGF is slightly higher than that from CLAD mixtures, which could be ascribed to stronger molecular interactions with the high surface area nanocellulose.

## Figures and Tables

**Table 1:** Structure and physicochemical properties of FFA.

FFA	Properties
Structure	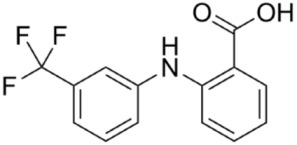
IUPAC Name	2-{[3-(trifluoromethyl)phenyl]amino}benzoic acid
Molar mass, g/mol	281
T melting, °C	134
pKa	3.9
logP	5.25

IUPAC = International Union of Pure and Applied Chemistry.

**Table 2:** FFA melting enthalpies in mixtures with MCC and CLAD. Results are presented as averages of three measurements with the standard deviation.

	T_on_, °C	T_m_, °C	ΔH, J/g _mix_	CrI *, %
FFA	134.4 ± 1.2	134.1 ± 0.0	94.1 ± 7.7	100
**FFA-MCC-N**				
*Polymorph A*	133.4 ± 0.0	134.5 ± 0.0	91.4 ± 7.1	97.2
**FFA-MCC-H**				
*Polymorph A*	133.2 ± 0.0	134.1 ± 0.1	45.8 ± 1.7	48.7
*Polymorph B*	128.1 ± 0.0	128.4 ± 0.1	18.0 ± 0.4	19.1
*Polymorph C*	125.5 ± 0.0	126.1 ± 0.0	6.3 ± 0.6	6.7
*Polymorph D*	121.9 ± 0.0	123.7 ± 0.2	13.0 ± 0.2	13.8
**FFA-CLAD-N**				
*Polymorph A*	133.5 ± 0.0	134.4 ± 0.1	11.2 ± 3.8	11.9
**FFA-CLAD-H**				
*Polymorph A*	131.3 ± 0.1	134.9 ± 1.3	0.07 ± 0.0	0.07

* CrI=Crystallinity Index.

**Table 3:** FFA melting enthalpies in mixtures with MCC and CLAD. Results are presented as averages with the standard deviation in parenthesis (n = 3).

	T_on_, °C	T_m_, °C	ΔH, J/g _mix_	CrI *, %
FFA	134.4 ± 1.2	134.1 ± 0.0	94.1 ± 7.7	100
**FFA-MCC-N**				
*Polymorph A*	133.6	134.7	80.8	85.8
**FFA-MCC-H**				
*Polymorph A*	133.4 ± 0.1	134.1 ± 0.0	4.2 ± 0.1	4.5
*Plolymorph B*	128.1 ± 0.0	129.3 ± 0.0	84.0 ± 0.6	89.2
**FFA-CLAD-N**				
*Polymorph A*	133.7	134.6	4.3	4.5
**FFA-CLAD-H**				
*Polymorph A*	131.9	133.3	0.001	0.0

* CrI=Crystallinity Index.

## Abbreviations

BCS, Biopharmaceutical classification system; CLAD, *Cladophora* cellulose; CrI, Crystallinity index; DSC, differential scanning calorimetry; FaSIF, fasted state simulated intestinal fluid; FeSIF, fed state simulated intestinal fluid; FFA, flufenamic acid; FTIR, Fourier-transform infrared; IUPAC, International Union of Pure and Applied Chemistry; IP intellectual property; MCC, microcrystalline cellulose; SGF, simulated gastric fluid; XRD, X-Ray diffraction.
